# Structures and dynamics of the novel S1/S2 protease cleavage site loop of the SARS-CoV-2 spike glycoprotein

**DOI:** 10.1016/j.yjsbx.2020.100038

**Published:** 2020-10-05

**Authors:** Thomas Lemmin, David Kalbermatter, Daniel Harder, Philippe Plattet, Dimitrios Fotiadis

**Affiliations:** a DS3Lab, System Group, Department of Computer Sciences, ETH Zurich, CH-8092 Zürich, Switzerland; b Trkola Group, Institute for Virology, University of Zurich, CH-8057 Zürich, Switzerland; c Institute of Biochemistry and Molecular Medicine, and Swiss National Centre of Competence in Research (NCCR) TransCure, University of Bern, CH-3012 Bern, Switzerland; d Division of Experimental and Clinical Research, Vetsuisse Faculty, University of Bern, CH-3012 Bern, Switzerland

**Keywords:** Ab initio modelling, Coronavirus, Furin cleavage site, Molecular dynamics simulation, SARS-CoV-2, Spike glycoprotein

## Abstract

At the end of 2019, a new highly virulent coronavirus known under the name SARS-CoV-2 emerged as a human pathogen. One key feature of SARS-CoV-2 is the presence of an enigmatic insertion in the spike glycoprotein gene representing a novel multibasic S1/S2 protease cleavage site. The proteolytic cleavage of the spike at this site is essential for viral entry into host cells. However, it has been systematically abrogated in structural studies in order to stabilize the spike in the prefusion state. In this study, multi-microsecond molecular dynamics simulations and *ab initio* modeling were leveraged to gain insights into the structures and dynamics of the loop containing the S1/S2 protease cleavage site. They unveiled distinct conformations, formations of short helices and interactions of the loop with neighboring glycans that could potentially regulate the accessibility of the cleavage site to proteases and its processing. In most conformations, this loop protrudes from the spike, thus representing an attractive SARS-CoV-2 specific therapeutic target.

## Graphical abstract

**Figure:**
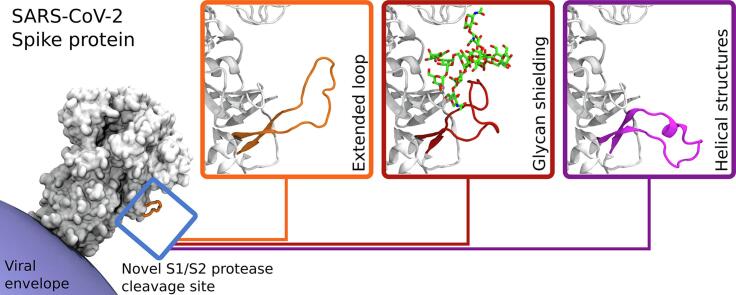

## 1 Introduction

Coronaviruses (CoVs) are a large group of enveloped, single-stranded positive-sense RNA viruses that infect humans and a wide range of animals including birds and mammals (Menachery et al., 2017). A new strain of coronavirus known as SARS-CoV-2 (severe acute respiratory syndrome coronavirus-2) and 2019-nCoV was first signaled at the end of 2019 in the Chinese city of Wuhan (Hubei province) as a human pathogen (Zhou et al., 2020b, Zhu et al., 2020). The SARS-CoV-2 coronavirus causes fever, a dry cough, breathing difficulties and in certain cases, pneumonia and severe respiratory syndrome, which can lead to death. This novel and highly infectious coronavirus respiratory illness was named COVID-19 (Chan et al., 2020, Huang et al., 2020) and marks in recent years the third emergence of a coronavirus that can be life threatening for humans. Previous coronavirus outbreaks include the SARS-CoV-1 and the Middle East Respiratory Syndrome (MERS), which appeared in 2003 (World Health Organization, 2003) and 2012 (World Health Organization, 2012), respectively. SARS-CoV-1 disappeared about two years later, whereas MERS continues to affect a small number of people, mainly in the Middle East. SARS-CoV-1 and -2, and MERS coronaviruses (MERS-CoV) are animal pathogens that crossed the species barriers and infected humans who had direct and indirect contact with infected animals (Lu et al., 2015). Unfortunately, SARS-CoV-2 can also be transmitted from human-to-human and has thus spread worldwide at an alarming rate. In March 2020, the World Health Organization (WHO) declared the worldwide outbreak of the new coronavirus as a pandemic. To date, no approved vaccines or proven therapeutics against coronaviruses infecting humans are available.

### 1.1 The spike glycoprotein – protein S

**Fig. 1:**
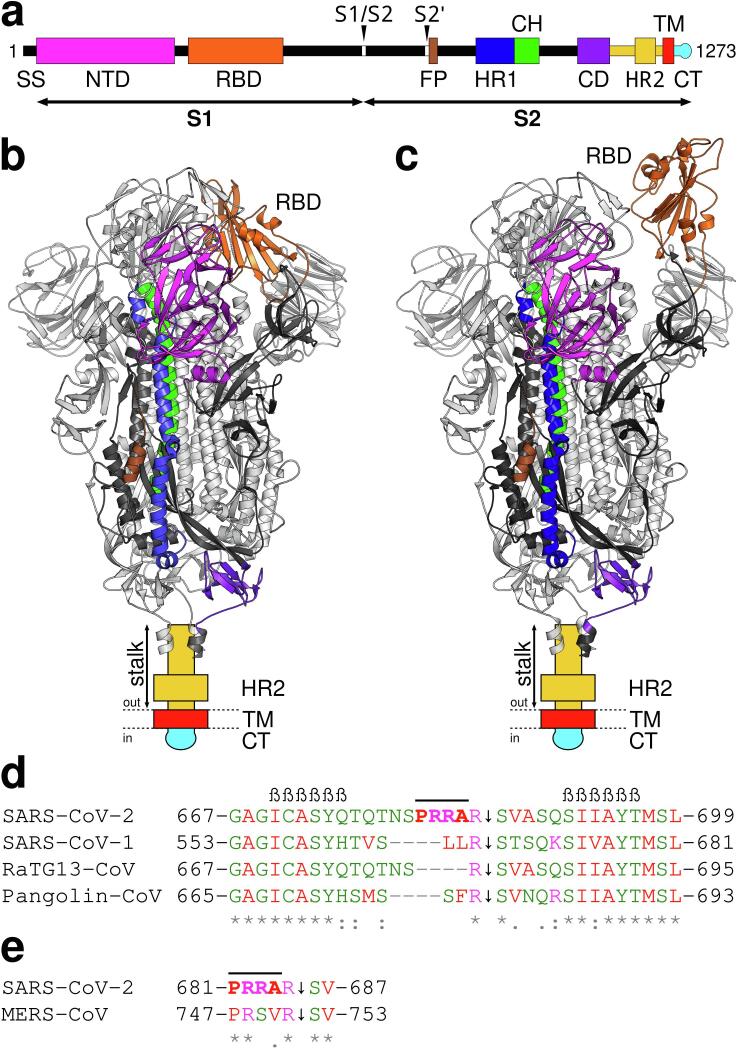
Structure of the SARS-CoV-2 S protein and comparison of S1/S2 protease cleavage site loops of closely related coronaviruses. **a** Schematic representation of the SARS-CoV-2 spike glycoprotein primary structure. The different domains are colored and defined as follows: SS, signal sequence; NTD, N-terminal domain; RBD, receptor-binding domain; S1/S2, S1/S2 protease cleavage site; S2′, S2′ protease cleavage site; FP, fusion peptide; HR1, heptad repeat (HR) 1; CH, central helix; CD, connector domain; HR2, stalk domain containing HR2; TM, transmembrane domain; CT, cysteine-rich cytoplasmic domain. Arrowheads mark protease cleavage sites S1/S2 and S2′. Prefusion state structures of the SARS-CoV-2 S protein ectodomain with: **b** all RBDs closed (PDB ID code: 6VXX) (Walls et al., 2020), and **c** one open and two closed RBDs (PDB ID code: 6VYB) (Walls et al., 2020). Domains of the spike glycoproteins for which no structural information is available are represented schematically. Glycans are not displayed. The domains are colored according to the color code given in panel **a**. Amino acid (aa) sequence alignment of selected CoV beta-hairpins containing S1/S2 protease cleavage site loops (**d**), and SARS-CoV-2 and MERS-CoV inner loop regions (**e**). The novel insertion identified in SARS-CoV-2 in the loop is highlighted with amino acid residues in bold and overlined. The aa residues of the two conserved beta-strands, which enclose the loop containing the S1/S2 priming site are indicated by β characters. The sequence conservation is represented with characters, i.e., positions that have a fully conserved residue (*), and conservation between groups of strongly (:) and weakly similar properties (·). Color coding of aa residues is according to their physicochemical properties: small and hydrophobic (including aromatic except tyrosine) are in red, acidic in blue, basic in magenta and others in green. Protease cleavage sites are indicated (↓). Sequence alignment was performed with Clustal Omega (Sievers et al., 2011) and the aa sequences of SARS-CoV-2 (GenBank: QHD43416.1), SARS-CoV-1 (GenBank: AYV99817.1), RaTG13-CoV (GenBank: QHR63300.2), Pangolin-CoV (GenBank: QIA48632.1) and MERS-CoV (GenBank: AFS88936.1).

The entry of coronaviruses into host cells is mediated by the spike glycoprotein (S protein) (Tortorici and Veesler, 2019), which is an important determinant for host range, cell tropism and pathogenicity of the virus (Lu et al., 2015). S proteins are type I glycoproteins and consist of about 1200 amino acids (aa), e.g., 1255 aa (SARS-CoV-1; GenBank: AYV99817.1) and 1273 aa (SARS-CoV-2; GenBank: QHD43416.1). The amino acid sequence identities of the bat RaTG13 and pangolin CoV S proteins to the SARS-CoV-2 S protein are ~97% (GenBank: QHR63300.2) and ~92% (GenBank: QIA48632.1), respectively. Spike glycoproteins form homotrimeric membrane protein complexes that protrude from the viral surface giving the viral particles a “crown” (corona)-like appearance. In contrast to other viruses, e.g., morbilliviruses such as measles virus and canine distemper virus (Plattet et al., 2016), which possess separate receptor and fusion proteins in the viral membrane, coronavirus spike proteins are composed of two functional domains/subunits: one acting as a receptor (S1) and the other as a fusion subunit (S2) (Fig. 1a).

S proteins can be divided into several conserved domains and motifs (Fig. 1a). The N-terminal S1 domain contains the receptor-binding domain (RBD), which recognizes the angiotensin-converting enzyme 2 as a receptor on host cells in SARS-CoV-1 (Li et al., 2003) and SARS-CoV-2 (Hoffmann et al., 2020b, Walls et al., 2020, Wrapp et al., 2020). The C-terminal S2 domain is responsible for mediating membrane fusion between the virus and the host cell, and includes the fusion peptide (FP), two heptad repeats (HR1 and HR2), the transmembrane domain (TM) and other domains (Fig. 1a).

### 1.2 Structure of the SARS-CoV-2 ectodomain S protein

At the beginning of 2020, structures of the SARS-CoV-2 S protein ectodomain trimer were published (Walls et al., 2020, Wrapp et al., 2020) providing valuable information on the complex architecture. It should be noted that the recombinant SARS-CoV-2 proteins were designed in a prefusion stabilized conformation, e.g., with an abrogated S1/S2 protease cleavage site. Cryo-electron microscopy (cryo-EM) of the SARS-CoV-2 S protein ectodomain structures in the closed state, where all RBDs are tightly packed together (Walls et al., 2020), and in the partially open state, with one open, two closed RBDs in the trimer (Walls et al., 2020, Wrapp et al., 2020) are available (Fig. 1b and c). Recently, the groups of McLellan (Hsieh et al., 2020) and Veesler (McCallum et al., 2020) presented additional engineered versions and structures of the SARS-CoV-2 S protein ectodomain stabilized with two open, one closed and all closed RBDs. The structure of SARS-CoV-2 S protein resembles that of SARS-CoV-1 (Walls et al., 2020, Wrapp et al., 2020). One difference consists in the packing of the RBDs in their closed conformations, i.e., the RBDs in SARS-CoV-1 are tightly packed against the N-terminal domain (NTD), while the S protein in SARS-CoV-2 is angled and closer to the central cavity of the trimer (Wrapp et al., 2020). Structures of SARS-CoV-2 resolved 48 (Walls et al., 2020) and 44 (Wrapp et al., 2020) of the 66 N-linked glycosylations per trimer (Wrapp et al., 2020).

### 1.3 The extended S1/S2 protease cleavage site of SARS-CoV-2 spike glycoprotein

Coronavirus spike glycoproteins are cleaved posttranslationally by host cell proteases into S1 and S2 domains, which remain bound via non-covalent interactions (Bosch et al., 2003). This processing step is known as priming and is essential for viral entry. In contrast to SARS-CoV-1, which contains a monobasic S1/S2 protease cleavage site that is processed upon entry into target cells, SARS-CoV-2 has an extended tribasic priming site including a pair of basic residues (Fig. 1d). Recent evidence from *in vitro* experiments reports that this tribasic site is not only recognized and cleaved by furin (Hoffmann et al., 2020a, Jaimes et al., 2020) but also by additional proteases (Jaimes et al., 2020). This tribasic protease cleavage site, hereinafter referred to as furin cleavage site, contains an insertion of 12 nucleotides coding the aa sequence 681-PRRA-684 (Fig. 1d) (Walls et al., 2020). The spike protein is thus already cleaved by furin or other proteases during biogenesis, differentiating this new virus from SARS-CoV-1 and other related CoVs (Walls et al., 2020). In coronaviruses, a second cleavage site called S2′ is localized upstream of the fusion peptide (Madu et al., 2009, Millet and Whittaker, 2015) (Fig. 1a). For full activation of the S protein and viral entry, cleavage at S1/S2 and S2′ is expected. Here again, the SARS-CoV-2 is markedly different. Unlike other CoVs, which exhibit a monobasic S2′ cleavage site (R↓S), SARS-CoV-2 and closely related bat CoVs display a dibasic cleavage site (KR↓SF) (Coutard et al., 2020). Interestingly, CoVs presenting monobasic cleavage sites appear to be less pathogenic to humans (Coutard et al., 2020).

Betacoronaviruses are divided into four lineages denoted as: A, B, C and D. The SARS-CoV-1 and the new SARS-CoV-2 are both part of lineage B, and the MERS-CoV is part of lineage C. With the exception of the recently emerged SARS-CoV-2, multibasic S1/S2 protease cleavage sites are totally absent in lineage B. Only the S protein from the MERS-CoV (lineage C) also contains a related dibasic cleavage site (Fig. 1e). Proteolytic cleavage at the S1/S2 site is essential for viral entry. Blocking of this event could reduce or inhibit viral entry. We therefore carried out an extensive investigation into the structures and dynamics of the loop containing this novel S1/S2 protease cleavage site (Fig. 1d).

## 2 Material and methods

### 2.1 Molecular dynamics simulations

Two 10-µs molecular dynamics (MD) simulations of the SARS-CoV-2 S protein under physiological conditions (aqueous solution, 310 K and 1 atm) were carried out by D. E. Shaw Research on their Anton2 supercomputers. These simulations are freely available and can be downloaded from the D. E. Shaw Research website (Shaw Research, 2020). The closed (6VXX, simulation 11021566) and the partially open (6VYB, simulation 11021571) structures were used as initial models. Missing loops were added and the structures were fully glycosylated. The final systems were solvated in an aqueous buffer and neutralized using NaCl ions at a concentration of 150 mM. The molecular dynamics simulations were carried out using the Amber force field (ff99SB-ILDN for the protein and general force field for the glycans) and the trajectory was saved every 1.2 ns.

### 2.2 Structural analyses

A principal component analysis (PCA) of the molecular dynamics simulations was performed using ProDy (Bakan et al., 2011). The k-means clustering was implemented with the scikit-learn package (Pedregosa et al., 2011) and the optimal number of clusters was determined using the Silhouette method (Rousseeuw, 1987).

### 2.3 Rosetta loop modeling

The missing loop containing the tribasic protease cleavage site was modeled using the remodel procedure in Rosetta (Huang et al., 2011). The procedure generated 600 loop conformations starting from the closed structure (6VXX). The model with the lowest energy was then further refined by running 600 instances of the Kinetic Closure (KIC) protocol in Rosetta (Mandell et al., 2009). The structures were clustered using the cluster application in Rosetta with a 2 Å radius and sorted by energy. We considered the top 10 clusters and removed the singletons.

## 3 Results and discussion

### 3.1 Structures and dynamics of the S1/S2 protease cleavage site loop in SARS-CoV-2 S protein

**Fig. 2:**
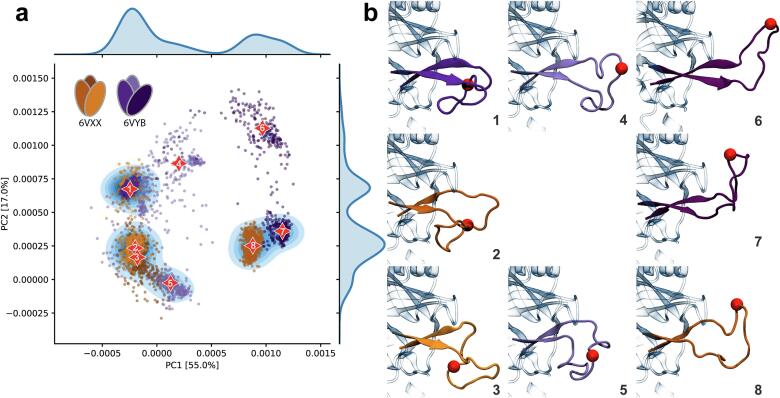
Dynamics of the loop containing the furin cleavage site in SARS-CoV-2 S protein. **a** Projection of the molecular dynamics simulations into the eigenspace formed by the two first components of a principal component (PC) analysis. Each point represents a conformation of the loop and the color indicates to which protomer and structure it belongs to: tones of orange, closed structure (6VXX) and tones of purple, partially open structure (6VYB). The marginal distributions are obtained using a kernel density estimate. The centroids from a k-means clustering are shown with red stars, and the corresponding structures in **b**. Representative conformations of the loop for each centroid are colored and labeled according the code defined in panel **a**. The red sphere indicates the furin cleavage site (Cα of R685).

In CoVs, two conserved beta-strands form an anti-parallel beta-sheet connected by a loop, which contains the S1/S2 protease cleavage site (Fig. 1d). In order to gain insights into the structures and dynamics of the loop containing the novel multibasic furin cleavage site of the SARS-CoV-2 spike glycoprotein, we analyzed two multi-microsecond molecular dynamics (MD) simulations made freely available by D.E. Shaw Research (Shaw Research, 2020). These simulations were initiated from the closed (6VXX) and partially open (6VYB) structures with modeled missing loops. A principal component analysis of the conformations sampled by the beta-hairpin containing the furin cleavage site (I670 to T696) shows that the loop samples several distinct conformations (Fig. 2a). A system is considered ergodic, if the time average equals the ensemble average. Despite of the significant simulation time, i.e., multi-microsecond MD simulations, the conformations sampled by each protomer are distinct (Fig. 2), thus indicating that ergodicity was not achieved.

**Fig. 3:**
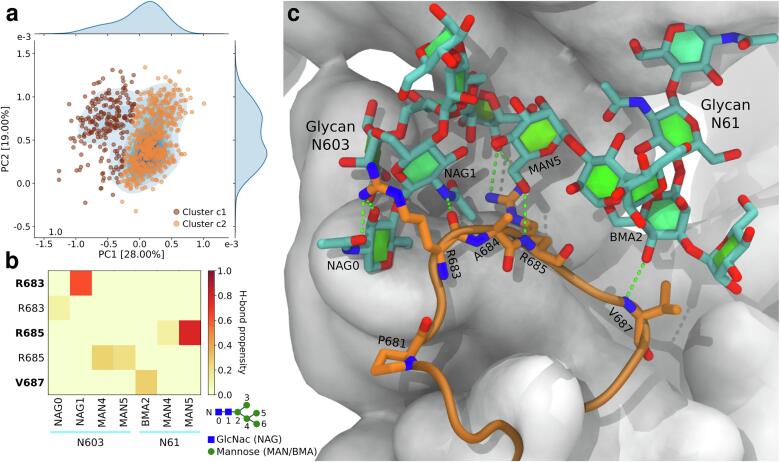
Interactions between the loop containing the furin cleavage site and neighboring glycans (N61 and N603). **a** Projection of the structures from cluster 8 (Fig. 2) into the eigenspace formed by the two first components of a principal component (PC) analysis. Each point represents a conformation of the loop and both glycans. The color indicates to which cluster it belongs. The marginal distributions are obtained using a kernel density estimate. **b** Heatmap of the propensity to form hydrogen bonds between the loop and each glycan. Labels in bold indicate interactions with the residue’s backbone. Glycan moieties are numbered according the schematic at the bottom right. Abbreviations used: GlcNac/NAG, N-acetylglucosamine; MAN, mannose; BMA, β-mannose. **c** Atomic representation of the centroid of cluster c2. Cartoon representation is used for the loop containing the furin cleavage site (orange). Interacting residues and the glycans are shown with orange and cyan sticks, respectively. The rest of the S-protein is represented as a white surface.

In order to isolate representative conformations, a k-means clustering was carried out in the eigenspace defined by the first three principal components, which account for 81% of the total variance. The optimal number of clusters (k = 8) was determined using the Silhouette method (Rousseeuw, 1987). During the MD simulations, the loop appears largely unstructured and samples several conformations extending outwards (Fig. 2b, clusters 4 – 7), making them potentially accessible for proteolytic cleavage. However, in three clusters, the loop also folds back towards the protein (Fig. 2b, clusters 1 – 3), causing the furin cleavage site to be less accessible. Finally, in one of the clusters of the closed structure (cluster 8), the loop points towards the apex and interacts extensively with the neighboring N-glycans (N61 and N603). The interactions remain stable throughout the simulations. A principal component analysis of conformations sampled by the beta-hairpin containing the furin cleavage site and the glycan rings indicates two comparable interaction modes (Fig. 3). The structures from cluster c1 were all sampled during the first 1 μs of simulation, thus corresponding to the initial equilibration of the interactions. We therefore focused the glycan analysis on the cluster c2. Two arginine residues (R683 and R685) dominate the interactions with the glycans and form a persistent network of hydrogen bonds with several glycan moieties (Fig. 3b and c). The backbone of V687 also interacted with N61 β-mannose in about 30% of the conformations. These glycans could thus play an important role in regulating the accessibility of the furin cleavage site. Taken together, these observations indicate a complex interplay between the dynamics of the novel multibasic S1/S2 protease cleavage site loop and neighboring glycans.

**Fig. 4:**
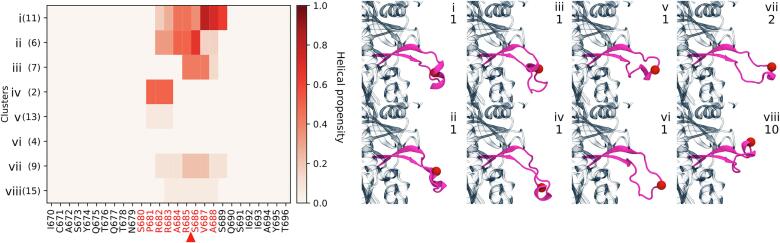
*Ab initio* modeling of the loop containing the furin cleavage site in S protein of SARS-CoV-2. **Left panel:** Heatmap indicating the helical propensity for each residue in a cluster. The clusters i-viii are sorted based on their energy score (most favorable first) and the number in parenthesis indicates the number of structures within a cluster. Residues in the protease cleavage site region are displayed in red. The furin cleavage site is indicated by a red arrowhead. **Right panel:** The lowest energy model with a helical structure for each cluster is shown. The upper and lower numbers indicate the cluster number and the rank of the model within the cluster, respectively. The red sphere shows the furin cleavage site (Cα of R685).

Since ergodicity was not achieved during the MD simulations, we used the *ab initio* modeling procedure of Rosetta (Huang et al., 2011), a powerful protein modeling software, to more extensively sample the conformations of the S1/S2 cleavage site containing loop. No noticeable energy gap was observed between the different *ab initio* models; thus they were first clustered. Singletons were removed from the ten lowest energy clusters, yielding up to eight clusters for the analysis (Fig. 4). The presence of a helical structure is observed for several of the low energy structures in most clusters and is formed in the vicinity of the furin cleavage site (Fig. 4). Such conformations, however, were never sampled during the MD simulations. The conformations from the MD simulation most similar to the *ab initio* models superposed with an average RMSD of 2.5 ± 0.2 Å. With the exception of one model, all the other *ab initio* models (Fig. 4) were closest to MD conformations that belonged to cluster 3 (Fig. 2). Only the MD conformation closest to model iv belonged to cluster 2. The presence of a helix could also influence the binding of the protease and thus cleavage of the loop containing the novel multibasic S1/S2 cleavage site.

### 3.2 Analysis of amino acid residues in the SARS-CoV-2 spike glycoprotein S1/S2 cleavage site containing loop, and of their potential structural and functional roles

From a structural point of view, the SARS-CoV-2 S protein proline residue (P681; Figs. 1d and 3c) in the insertion is eye-catching, because of the special and unique structural properties of this proteinogenic amino (imino) acid. MERS-CoV S protein is one of the other rare CoV spike proteins, that also contains a proline residue at the corresponding position in the S1/S2 protease cleavage site (Fig. 1e). When searching the database FurinDB (Tian et al., 2011) (http://www.nuolan.net/substrates.html), which includes experimentally verified furin cleavage sites, it appears that a proline residue at position P5, i.e., the 5th residue prior to the furin cleavage site, is rare and appears in only 5 out of 132 sequences (three mammalian and two viral sequences). Since proline is unable to adopt several main chain conformations in proteins, it imposes strong conformational restraints on the peptide chain. It is therefore often found in turns, which force the peptide chain to change directions and separate secondary structures. This is supported by the *ab initio* modeling, where the proline is found at the N-terminus of short helices in several models (Fig. 4). Finding this proline in the insertion, just before basic amino acid residues, which define the SARS-CoV-2 S protein furin cleavage site is interesting, since it nicely separates the cleavage site from other structural elements, which might better expose it to the proteases. Recently, Andersen *et al.* (Andersen et al., 2020) proposed that the presence of the proline residue in the insertion would result in the addition of O-linked glycans at flanking positions S673, T678 and S686. In the recent structures of the S protein ectodomain (Walls et al., 2020, Wrapp et al., 2020), only S673 could be modeled into the density map from cryo-EM. The authors of the published structures did not model a glycan at position S673 and no additional density is visible near S673 when inspecting the density maps (https://www.ebi.ac.uk/pdbe/emdb/; EMD-21452 (Walls et al., 2020), EMD-21457 (Walls et al., 2020) and EMD-21375 (Wrapp et al., 2020)). Glycans on the surface of viral proteins often mask immunodominant epitopes, thus protecting them from the host’s immune system. However, glycosylation of residues flanking the furin cleavage site does not appear to be beneficial, since this would prevent the full maturation of the S protein by shielding the cleavage site from the proteases. In addition and more important, the recent mapping of O-glycosylation in SARS-CoV-2 spike protein by high resolution LC-MS/MS does not report O-glycosylation at positions S673, T678 and S686 (Shajahan et al., 2020).

The presence of alanine (A684) at position P2, i.e., the 2nd residue prior to the furin cleavage site, is also unusual in a furin cleavage site and appears in only 5 out of 132 sequences in the FurinDB (Tian et al., 2011). This position (P2) is predominantly occupied by Arg or Lys, and it has been shown that a basic residue at P2 greatly enhances processing efficiency (Shiryaev et al., 2013, Thomas, 2002). Thus, the alanine at P2 in SARS-CoV-2 S protein is expected to decrease the furin cleavage efficiency compared to sites containing basic amino acids at P2. However, this reduction in cleavage efficiency might be largely compensated by the presence of a total of three basic residues (i.e., a relatively high number in SARS-CoV-2 compared to other CoVs, see Fig. 1d) at the S1/S2 protease cleavage site loop.

### 3.3 Potential function of the furin cleavage site in SARS-CoV-2 S protein

From a functional point of view, the insertion of a multibasic protease cleavage site at S1/S2 in SARS-CoV-2 is an important new feature, which may account for its increased virulence. The highly pathogenic avian influenza (AI) viruses are known to have evolved from low-pathogenic AI viruses (Perdue et al., 1997). While low-pathogenic AI viruses contain a single arginine residue, highly pathogenic AI viruses contain multiple basic amino acid (aa) residues at the cleavage site of the surface glycoprotein hemagglutinin (Chen et al., 1998, Ito et al., 2001, Perdue et al., 1997). Incorporation of basic aa at these sites was proposed to have originated by mutation/recombination events in influenza H9 viruses (Lee and Whittaker, 2017), or by polymerase slippage in influenza H5 and possibly H7 viruses (Nao et al., 2017). A site containing a single arginine is cleaved by trypsin-like proteases, whereas multiple basic amino acids are recognized by several cellular proteases including furin (Chen et al., 1998, Nao et al., 2017). Such characteristics may also contribute to understanding the differences of how SARS-CoV-1 (monobasic S1/S2 cleavage site) and SARS-CoV-2 (tribasic S1/S2 cleavage site) infect humans. Here, the 681-PRRA-684 insert (Fig. 1d) may not only confer an advantage in SARS-CoV-2 cell entry, but may consequently facilitate human-to-human transmission and thus the rapid spread of the disease compared to CoVs without a multibasic S1/S2 protease cleavage site.

An additional feature that will influence the protease cleavage efficiency at the S1/S2 site of CoVs, is the length of the loop containing this site, which is flanked by two conserved beta-strands (Fig. 1d, also Fig. 2, Fig. 4 for structures). In SARS-CoV-2, the loop harboring the S1/S2 cleavage site, has a length of 15 aa and is the longest when compared with closely related CoVs, which have all 11 aa (Fig. 1d). Recently, a novel bat isolate (RmYN02), which exhibits a nucleotide sequence identity of about 93% with the SARS-CoV-2 genome, was identified (Zhou et al., 2020a). Conversely, the loop containing the S1/S2 protease cleavage site of the RmYN02 S protein is relatively short containing only 9 aa (Fig. 2H in (Zhou et al., 2020a)) compared to SARS-CoV-2 (15 aa) and closely related CoVs (11 aa) (Fig. 1d).

## 4 Conclusion

The novel multibasic S1/S2 protease cleavage site is an important new feature of SARS-CoV-2 and represents an attractive therapeutic target, since viral entry could be reduced or inhibited by blocking the proteolytic cleavage event. Our analyses of molecular dynamic simulations and *ab initio* modeling showed that the loop containing this cleavage site protrudes from the S protein surface, making it accessible to proteases. The neighboring N-linked glycans might, however, modulate accessibility of the protease cleavage site. The *ab initio* modeling also indicated that the loop might be moderately structured forming short helices close to the cleavage site. The impact of the nature, length, structure and dynamics of this loop on protease cleavage efficiency, and ultimately, the overall pathogenicity of CoVs remains, however, an open question that warrants immediate detailed analysis due to the current pandemic crisis.

## CRediT authorship contribution statement

**Thomas Lemmin:** Conceptualization, Methodology, Software, Data curation, Formal analysis, Investigation, Resources, Visualization, Writing - original draft, Writing - review & editing, Funding acquisition. **David Kalbermatter:** Formal analysis, Investigation, Visualization, Writing - review & editing. **Daniel Harder:** Formal analysis, Investigation, Writing - review & editing. **Philippe Plattet:** Conceptualization, Writing - original draft, Writing - review & editing, Funding acquisition. **Dimitrios Fotiadis:** Conceptualization, Writing - original draft, Writing - review & editing, Supervision, Project administration, Funding acquisition.

## Declaration of Competing Interest

The authors declare that they have no known competing financial interests or personal relationships that could have appeared to influence the work reported in this paper.

## References

[b0005] Andersen K.P., Rambaut A., Lipkin W.I., Holmes E.C., Garry R.F. (2020). The proximal origin of SARS-CoV-2. Nat. Med..

[b0010] Bakan A., Meireles L.M., Bahar I. (2011). ProDy: protein dynamics inferred from theory and experiments. Bioinformatics.

[b0015] Bosch B.J., van der Zee R., de Haan C.A., Rottier P.J. (2003). The coronavirus spike protein is a class I virus fusion protein: structural and functional characterization of the fusion core complex. J. Virol..

[b0020] Chan J.F., Yuan S., Kok K.H., To K.K., Chu H., Yang J., Xing F., Liu J., Yip C.C., Poon R.W., Tsoi H.W., Lo S.K., Chan K.H., Poon V.K., Chan W.M., Ip J.D., Cai J.P., Cheng V.C., Chen H., Hui C.K., Yuen K.Y. (2020). A familial cluster of pneumonia associated with the 2019 novel coronavirus indicating person-to-person transmission: a study of a family cluster. Lancet.

[b0025] Chen J., Lee K.H., Steinhauer D.A., Stevens D.J., Skehel J.J., Wiley D.C. (1998). Structure of the hemagglutinin precursor cleavage site, a determinant of influenza pathogenicity and the origin of the labile conformation. Cell.

[b0030] Coutard B., Valle C., de Lamballerie X., Canard B., Seidah N.G., Decroly E. (2020). The spike glycoprotein of the new coronavirus 2019-nCoV contains a furin-like cleavage site absent in CoV of the same clade. Antiviral Res..

[b0035] Hoffmann M., Kleine-Weber H., Pohlmann S. (2020). A multibasic cleavage site in the spike protein of SARS-CoV-2 is essential for infection of human lung cells. Mol. Cell.

[b0040] Hoffmann M., Kleine-Weber H., Schroeder S., Kruger N., Herrler T., Erichsen S., Schiergens T.S., Herrler G., Wu N.H., Nitsche A., Muller M.A., Drosten C., Pohlmann S. (2020). SARS-CoV-2 cell entry depends on ACE2 and TMPRSS2 and is blocked by a clinically proven protease inhibitor. Cell.

[b0045] Hsieh C.L., Goldsmith J.A., Schaub J.M., DiVenere A.M., Kuo H.C., Javanmardi K., Le K.C., Wrapp D., Lee A.G., Liu Y., Chou C.W., Byrne P.O., Hjorth C.K., Johnson N.V., Ludes-Meyers J., Nguyen A.W., Park J., Wang N., Amengor D., Lavinder J.J., Ippolito G.C., Maynard J.A., Finkelstein I.J., McLellan J.S. (2020). Structure-based design of prefusion-stabilized SARS-CoV-2 spikes. Science.

[b0050] Huang C., Wang Y., Li X., Ren L., Zhao J., Hu Y., Zhang L., Fan G., Xu J., Gu X., Cheng Z., Yu T., Xia J., Wei Y., Wu W., Xie X., Yin W., Li H., Liu M., Xiao Y., Gao H., Guo L., Xie J., Wang G., Jiang R., Gao Z., Jin Q., Wang J., Cao B. (2020). Clinical features of patients infected with 2019 novel coronavirus in Wuhan, China. Lancet.

[b0055] Huang P.S., Ban Y.E., Richter F., Andre I., Vernon R., Schief W.R., Baker D. (2011). RosettaRemodel: a generalized framework for flexible backbone protein design. PLoS ONE.

[b0060] Ito T., Goto H., Yamamoto E., Tanaka H., Takeuchi M., Kuwayama M., Kawaoka Y., Otsuki K. (2001). Generation of a highly pathogenic avian influenza A virus from an avirulent field isolate by passaging in chickens. J. Virol..

[b0065] Jaimes, J.A., Millet, J.K., Whittaker, G.R., 2020. Proteolytic cleavage of the SARS-CoV-2 spike protein and the role of the novel S1/S2 site. iScience 23, 101212. 10.1016/j.isci.2020.101212 PMC7255728 32512386

[b0070] Lee D.W., Whittaker G.R. (2017). Use of AAScatterPlot tool for monitoring the evolution of the hemagglutinin cleavage site in H9 avian influenza viruses. Bioinformatics.

[b0075] Li W., Moore M.J., Vasilieva N., Sui J., Wong S.K., Berne M.A., Somasundaran M., Sullivan J.L., Luzuriaga K., Greenough T.C., Choe H., Farzan M. (2003). Angiotensin-converting enzyme 2 is a functional receptor for the SARS coronavirus. Nature.

[b0080] Lu G., Wang Q., Gao G.F. (2015). Bat-to-human: spike features determining 'host jump' of coronaviruses SARS-CoV, MERS-CoV, and beyond. Trends Microbiol..

[b0085] Madu I.G., Roth S.L., Belouzard S., Whittaker G.R. (2009). Characterization of a highly conserved domain within the severe acute respiratory syndrome coronavirus spike protein S2 domain with characteristics of a viral fusion peptide. J. Virol..

[b0090] Mandell D.J., Coutsias E.A., Kortemme T. (2009). Sub-angstrom accuracy in protein loop reconstruction by robotics-inspired conformational sampling. Nat. Methods.

[b0095] McCallum M., Walls A.C., Bowen J.E., Corti D., Veesler D. (2020). Structure-guided covalent stabilization of coronavirus spike glycoprotein trimers in the closed conformation.

[b0100] Menachery V.D., Graham R.L., Baric R.S. (2017). Jumping species-a mechanism for coronavirus persistence and survival. Curr. Opin. Virol..

[b0105] Millet J.K., Whittaker G.R. (2015). Host cell proteases: Critical determinants of coronavirus tropism and pathogenesis. Virus Res..

[b0110] Nao N., Yamagishi J., Miyamoto H., Igarashi M., Manzoor R., Ohnuma A., Tsuda Y., Furuyama W., Shigeno A., Kajihara M., Kishida N., Yoshida R., Takada A. (2017). Genetic predisposition to acquire a polybasic cleavage site for highly pathogenic avian influenza virus hemagglutinin..

[b0115] Pedregosa F., Varoquaux G., Gramfort A., Michel V., Thirion B., Grisel O., Blondel M., Prettenhofer P., Weiss R., Dubourg V., Vanderplas J., Passos A., Cournapeau D., Brucher M., Perrot M., Duchesnay E., Louppe G. (2011). Scikit-learn: Machine learning in Python. J. Mach. Learn. Res..

[b0120] Perdue M.L., Garcia M., Senne D., Fraire M. (1997). Virulence-associated sequence duplication at the hemagglutinin cleavage site of avian influenza viruses. Virus Res..

[b0125] Plattet P., Alves L., Herren M., Aguilar H.C. (2016). Measles virus fusion protein: structure, function and inhibition. Viruses.

[b0130] Rousseeuw P.J. (1987). Silhouettes: A graphical aid to the interpretation and validation of cluster analysis. J. Comput. Appl. Math..

[b0135] Shajahan A., Supekar N.T., Gleinich A.S., Azadi P. (2020). Deducing the N- and O- glycosylation profile of the spike protein of novel coronavirus SARS-CoV-2. Glycobiology.

[b0140] Shaw Research, D.E., 2020. Molecular dynamics simulations related to SARS-CoV-2. D. E. Shaw Research Technical Data http://www.deshawresearch.com/resources_sarscov2.html.

[b0145] Shiryaev S.A., Chernov A.V., Golubkov V.S., Thomsen E.R., Chudin E., Chee M.S., Kozlov I.A., Strongin A.Y., Cieplak P. (2013). High-resolution analysis and functional mapping of cleavage sites and substrate proteins of furin in the human proteome. PLoS ONE.

[b0150] Sievers F., Wilm A., Dineen D., Gibson T.J., Karplus K., Li W., Lopez R., McWilliam H., Remmert M., Soding J., Thompson J.D., Higgins D.G. (2011). Fast, scalable generation of high-quality protein multiple sequence alignments using Clustal Omega. Mol. Syst. Biol..

[b0155] Thomas G. (2002). Furin at the cutting edge: from protein traffic to embryogenesis and disease. Nat. Rev. Mol. Cell Biol..

[b0160] Tian S., Huang Q., Fang Y., Wu J. (2011). FurinDB: A database of 20-residue furin cleavage site motifs, substrates and their associated drugs. Int. J. Mol. Sci..

[b0165] Tortorici M.A., Veesler D. (2019). Structural insights into coronavirus entry. Adv. Virus Res..

[b0170] Walls A.C., Park Y.J., Tortorici M.A., Wall A., McGuire A.T., Veesler D. (2020). Structure, function, and antigenicity of the SARS-CoV-2 spike glycoprotein. Cell.

[b0175] World Health Organization, W., 2003. Severe Acute Respiratory Syndrome CoronaVirus-1 (SARS-CoV-1), https://www.who.int/csr/sars/en/.

[b0180] World Health Organization, W., 2012. Middle East Respiratory Syndrome CoronaVirus (MERS-CoV), https://www.who.int/emergencies/mers-cov/en/.

[b0185] Wrapp D., Wang N., Corbett K.S., Goldsmith J.A., Hsieh C.L., Abiona O., Graham B.S., McLellan J.S. (2020). Cryo-EM structure of the 2019-nCoV spike in the prefusion conformation. Science.

[b0190] Zhou H., Chen X., Hu T., Li J., Song H., Liu Y., Wang P., Liu D., Yang J., Holmes E.C., Hughes A.C., Bi Y., Shi W. (2020). A novel bat coronavirus closely related to SARS-CoV-2 contains natural insertions at the S1/S2 cleavage site of the spike protein. Curr. Biol..

[b0195] Zhou P., Yang X.L., Wang X.G., Hu B., Zhang L., Zhang W., Si H.R., Zhu Y., Li B., Huang C.L., Chen H.D., Chen J., Luo Y., Guo H., Jiang R.D., Liu M.Q., Chen Y., Shen X.R., Wang X., Zheng X.S., Zhao K., Chen Q.J., Deng F., Liu L.L., Yan B., Zhan F.X., Wang Y.Y., Xiao G.F., Shi Z.L. (2020). A pneumonia outbreak associated with a new coronavirus of probable bat origin. Nature.

[b0200] Zhu, N., Zhang, D., Wang, W., Li, X., Yang, B., Song, J., Zhao, X., Huang, B., Shi, W., Lu, R., Niu, P., Zhan, F., Ma, X., Wang, D., Xu, W., Wu, G., Gao, G.F., Tan, W., China Novel Coronavirus, I., Research, T., 2020. A novel coronavirus from patients with pneumonia in China, 2019. N. Engl. J. Med. 382, 727-733. 10.1056/NEJMoa2001017 PMC7092803 31978945

